# Supplementary material for: In Situ Processing and Efficient Environmental Detection (iSPEED) of tree pests and pathogens using point-of-use real-time PCR
Source: PLoS One. 2020 Apr 2;15(4):e0226863. doi: 10.1371/journal.pone.0226863 (PMC7117680; doi:10.1371/journal.pone.0226863)
Supplement: S1 Table — The table lists all the oligonucleotides used in the various tests. Probes were ordered with a FAM fluorophore for simplex reactions and with the FAM and CY5 combination for duplexes. The + sign denotes a LNA base. (DOCX) [file pone.0226863.s001.docx]

**S1 Table. Oligonucleotide sequences used in qPCR assays.** The table lists all the oligonucleotides used in the various tests. Probes were ordered with a FAM fluorophore for simplex reactions and with the FAM and CY5 combination for duplexes. The + sign denotes a LNA base.

| **Name** | **Type** | **Assay** | **Sequence** | **Reference** |
| --- | --- | --- | --- | --- |
| Pram-C62-F | Primer | *P. ramorum*, TAIGA-C62  nuclear | AACATGCTCGTGCTCAAGTG | [(1)](https://www.zotero.org/google-docs/?wNfyLp) |
| Pram-C62-R | Primer |  | CGGTGTTCTGGCGTTCTAGT |  |
| Pram-C62-P | Probe |  | CAAGGGGACCGGAACCGTAT |  |
| Phyto_gen_Fflap | Primer | *P. ramorum*, *TrnM*  mitochondrial | AATAAATCATAACCTTCTTTACAACAAGAATTAATGAG | [(2)](https://www.zotero.org/google-docs/?DaC4Vg) |
| Pram_Rflap | Primer |  | AATAAATCATAATATAGGTAAAATTTGTAATAAATGTTGACT |  |
| Pram_nad9sp_1F | Probe |  | ACGTTACGTCTAGACTTGTATTATGCATTG |  |
| FS1 F2-16 | Primer | *Lymantria dispar* | GATGGTGGGTGTCGT | [(3)](https://www.zotero.org/google-docs/?sHOGPM) |
| FS1 R176-200 (71–95) | Primer |  | GATTCATCTGATCCTGATAATTCAT |  |
| FS1 AGM 62–86 | Probe |  | ACTCAACATAAAGTATGCCAACTCG |  |
| FS1 EGM RC 21–47 | Probe |  | AGTACTGCTGTATACATTTTAAACGTC |  |
| Cronartium Dcon10 1 F | Primer | *C. comandrae* | AAAGCACTCACCCTTCGGTC | This work, unpublished results |
| Cronartium Dcon10 1 R | Primer |  | GACTGGACACATTGGCTCCTT |  |
| Cribicola Dcon10 1 | Probe |  | TGGCC+T+C+G+ACGTGT |  |
| Ccomandrae Dcon10 1 | Probe |  | TGGC+C+T+T+C+ACGTGT |  |
| Smus_SSU193F | Primer | *S. musiva* | CGGTATTTTCAGCCTGCAG | [(4)](https://www.zotero.org/google-docs/?LmlBLl) |
| Smus_SSU_288R | Primer |  | GCCGTTATCCGTACAACTGA |  |
| Smus_SSU243F_probe | Probe |  | AGGTAGATATTAGGACAATTGGTGTAAAGATGAT |  |
| rbcL_delto-1F | Primer | *Populus spp.* | CACCCTTGGGGAAATGC |  |
| rbcL_delto-1R | Primer |  | CTTCACGGATAATTTCATTACCCTC |  |
| rbcL_delto_probe | Probe |  | CGAGTAGCTCTAGAAGCATGTGTACAAGCT |  |
