# Supplementary material for: In Situ Processing and Efficient Environmental Detection (iSPEED) of tree pests and pathogens using point-of-use real-time PCR
Source: PLoS One. 2020 Apr 2;15(4):e0226863. doi: 10.1371/journal.pone.0226863 (PMC7117680; doi:10.1371/journal.pone.0226863)
Supplement: S2 Table — The table lists the reagents used to prepare the fresh reactions for each assay. (DOCX) [file pone.0226863.s002.docx]

**S2 Table. Reagents used in fresh reactions.** The table lists the reagents used to prepare the fresh reactions for each assay.

| **Target organism** | **Reagent** | **Amount** |
| --- | --- | --- |
| *Sphaerulina musiva* | DNA template | 2 µL |
|  | QuantiTect mastermix | 10 µL |
|  | Primer 10 µM, each | 0.8 µL |
|  | Probe 100 µM | 4 µL |
|  | Trehalose 30% | 1.13 µL |
|  | Water | 1.27 µL |
|  | Total volume | 20 µL |
| *Cronartium* spp.^1^ | DNA template | 2 µL |
|  | QuantiTect mastermix | 10 µL |
|  | 20x Primers/probe mix | 1 µL |
|  | Trehalose 30% | 1.22 µL |
|  | Water | 4.78 µL |
|  | Total volume | 20 µL |
| *Phytophthora ramorum*^2^ | DNA template | 2 µL |
|  | QuantiTect mastermix | 10 µL |
|  | 20x Primers/probe mix | 1 µL |
|  | Trehalose 30% | 1.22 µL |
|  | Water | 5.78 µL |
|  | Total volume | 20 µL |
| *Lymantria dispar* | DNA template | 2 µL |
|  | QuantiTect mastermix | 10 µL |
|  | Primer 100 µM, each | 0.1 µL |
|  | Probe 100 µM, each | 0.04 µL |
|  | Trehalose 30% | 1.14 µL |
|  | Water | 6.58 µL |
|  | Total volume | 20 µL |
| ^1^The 20x Primers/probe mix is prepared to 5 µM for each primer and 2 µM for each probe | | |
| ^2^The 20x Primers/probe mix is prepared to 4 µM for each primer and the probe | | |
