# Supplementary material for: In Situ Processing and Efficient Environmental Detection (iSPEED) of tree pests and pathogens using point-of-use real-time PCR
Source: PLoS One. 2020 Apr 2;15(4):e0226863. doi: 10.1371/journal.pone.0226863 (PMC7117680; doi:10.1371/journal.pone.0226863)
Supplement: S3 Table — The table lists the reagents used to prepare the lyophilized strips for each assay. (DOCX) [file pone.0226863.s003.docx]

**S3 Table. Reagents used in lyophilized reactions.** The table lists the reagents used to prepare the lyophilized strips for each assay.

| **Target organism** | **Reagent** | **Amount** |
| --- | --- | --- |
| *Sphaerulina musiva* | QuantiTect mastermix | 10 µL |
|  | Primer 100 µM, each | 0.08 µL |
|  | Probe 100 µM | 0.04 µL |
|  | Trehalose 30%^1^ | 1.13 µL |
|  | Total volume | 11.33 µL |
| *Cronartium* spp.^2^ | QuantiTect mastermix | 10 µL |
|  | 20x Primers/probe mix | 1 µL |
|  | Trehalose 30%^1^ | 1.22 µL |
|  | Total volume | 12.22 µL |
| *Phytophthora ramorum*^3^ | QuantiTect mastermix | 10 µL |
|  | 20x Primers/probe mix | 1 µL |
|  | Trehalose 30%^1^ | 1.22 µL |
|  | Volume | 12.22 µL |
| *Lymantria dispar* | QuantiTect mastermix | 10 µL |
|  | Primer 100 µM, each | 0.1 µL |
|  | Probe 100 µM, each | 0.04 µL |
|  | Trehalose 30%^1^ | 1.14 µL |
|  | Total volume | 11.42 µL |
| ^1^For the reactions without trehalose, the 30% trehalose is omitted and the total aliquoted volume is adjusted accordingly. | | |
| ^2^The 20x Primers/probe mix is prepared to 5 µM for each primer and 2 µM for each probe. | | |
| ^3^The 20x Primers/probe mix is prepared to 4 µM for each primer and the probe | | |
