# Supplementary material for: In Situ Processing and Efficient Environmental Detection (iSPEED) of tree pests and pathogens using point-of-use real-time PCR
Source: PLoS One. 2020 Apr 2;15(4):e0226863. doi: 10.1371/journal.pone.0226863 (PMC7117680; doi:10.1371/journal.pone.0226863)
Supplement: S4 Table — DNA was extracted from leaves of poplar hybrids (P. trichocarpa x P. deltoides) infected by S. musiva using a Qiagen DNA extraction column and a field-ready protocol using Edwards buffer. DNA amplification was conducted in triplicate by qPCR using field-ready lyophilized and fresh reagents. Average Ct values of the replicates are reported for each of the conditions tested with the plant internal control (RbcL) and the S. musiva (SepMu) assays. All tests were conducted using material from the same leaf disc to allow direct comparisons between extraction methods. Both probes used carry the FAM fluorophore. (DOCX) [file pone.0226863.s004.docx]

**S4 Table. Real-time PCR amplification of *Sphaerulina musiva* directly from naturally-infected poplar hybrid leaves.** DNA was extracted from leaves of poplar hybrids (*P. trichocarpa* x *P. deltoides)* infected by *S. musiva* using a Qiagen DNA extraction column and a field-ready protocol using Edwards buffer. DNA amplification was conducted in triplicate by qPCR using field-ready lyophilized reagents and fresh reagents. Average C_t_ values of the replicates are reported for each of the conditions tested with the plant internal control (RbcL) and the *S. musiva* (SepMu) assays. All tests were conducted using material from the same leaf disc to allow direct comparisons between extraction methods. Both probes used carry the FAM fluorophore.

| **Target** | **Extraction** | **Reagents** | **C_t_ values** | **Standard dev.** | **Rep.** |
| --- | --- | --- | --- | --- | --- |
| RbcL | Column | Lyophilized | 17.52 | 0.03 | 3 |
|  |  | Fresh | 17.3 | 0.11 | 3 |
|  | Edward buffer | Lyophilized | 21.24 | 0.07 | 3 |
|  |  | Fresh | 21.46 | 0.08 | 3 |
| SepMu | Column | Lyophilized | 23.8 | 0.42 | 3 |
|  |  | Fresh | 23.34 | 0.07 | 3 |
|  | Edward buffer | Lyophilized | 25.12 | 0.02 | 3 |
|  |  | Fresh | 25.41 | 0.09 | 3 |
