# Supplementary material for: In Situ Processing and Efficient Environmental Detection (iSPEED) of tree pests and pathogens using point-of-use real-time PCR
Source: PLoS One. 2020 Apr 2;15(4):e0226863. doi: 10.1371/journal.pone.0226863 (PMC7117680; doi:10.1371/journal.pone.0226863)
Supplement: S5 Table — DNA was extracted from rhododendron leaves using a Qiagen DNA extraction column and a field-ready protocol using Edwards buffer. A pure culture of P. ramorum was also extracted with a Qiagen extraction protocol as a positive control. DNA amplification was conducted in triplicate by qPCR using field-ready lyophilized reagents and fresh reagents. Average Ct values of the replicates are reported for each of the conditions tested with mitochondrial and nuclear P. ramorum assays. All tests were conducted using material from the same leaf disc. Both probes used carry the FAM fluorophore. NA = No Amplification. (DOCX) [file pone.0226863.s005.docx]

**S5 Table. Real-time PCR amplification of *Phytophthora ramorum* from artificially infected *rhododendron* leaves.** DNA was extracted from rhododendron leaves using a Qiagen DNA extraction column and a field-ready protocol using Edwards buffer. A pure culture of *P. ramorum* was also extracted with a Qiagen extraction protocol as a positive control. DNA amplification was conducted in triplicate by qPCR using field-ready lyophilized reagents and fresh reagents. Average C_t_ values of the replicates are reported for each of the conditions tested with mitochondrial and nuclear *P. ramorum* assays. All tests were conducted using material from the same leaf disc. Both probes used carry the FAM fluorophore. NA = No Amplification.

| **Material** | **Extraction** | **Target** | **Reagents** | **C_t_ values** | **Standard dev.** | **Rep.** |
| --- | --- | --- | --- | --- | --- | --- |
| Culture | Column | *TrnM* mitochondrial | Fresh | 19.18 | 0.04 | 3 |
| *R. BCHM* | Edwards buffer |  |  | NA | NA | 3 |
| *R. macrophyllum* |  |  |  | NA | NA | 3 |
| *R. purdomii* |  |  |  | NA | NA | 3 |
| *R. BCHM*, inoculated |  |  |  | 32.16 | 0.05 | 3 |
| *R. macrophyllum*, inoculated |  |  |  | 28.16 | 0.44 | 3 |
| *R. purdomii*, inoculated |  |  |  | 26.64 | 0.07 | 3 |
| Culture | Column |  | Lyophilized | 15.77 | 0.13 | 3 |
| *R. BCHM* | Edwards buffer |  |  | NA | NA | 3 |
| *R. macrophyllum* |  |  |  | NA | NA | 3 |
| *R. purdomii* |  |  |  | NA | NA | 3 |
| *R. BCHM*, inoculated |  |  |  | 29.12 | 0.09 | 3 |
| *R. macrophyllum*, inoculated |  |  |  | 27.23 | 0.09 | 3 |
| *R. purdomii*, inoculated |  |  |  | 26.03 | 0.19 | 3 |
| Culture | Column | TAIGA-C62  Nuclear | Fresh | 25.80 | 0.10 | 3 |
| *R. BCHM* | Edwards buffer |  |  | NA | NA | 3 |
| *R. macrophyllum* |  |  |  | NA | NA | 3 |
| *R. purdomii* |  |  |  | NA | NA | 3 |
| *R. BCHM*, inoculated |  |  |  | 34.62 | 0.48 | 3 |
| *R. macrophyllum*, inoculated |  |  |  | 33.40 | 0.31 | 3 |
| *P. purdomii*, inoculated |  |  |  | 34.24 | 0.32 | 3 |
| Culture | Column |  | Lyophilized | 21.93 | 0.55 | 3 |
| *R. BCHM* | Edwards buffer |  |  | NA | NA | 3 |
| *R. macrophyllum* |  |  |  | NA | NA | 3 |
| *R. purdomii* |  |  |  | NA | NA | 3 |
| *R. BCHM*, inoculated |  |  |  | 33.48 | 0.07 | 3 |
| *R. macrophyllum*, inoculated |  |  |  | 33.20 | 0.24 | 3 |
| *R. purdomii*, inoculated |  |  |  | 33.65 | 0.26 | 3 |
