# Supplementary material for: In Situ Processing and Efficient Environmental Detection (iSPEED) of tree pests and pathogens using point-of-use real-time PCR
Source: PLoS One. 2020 Apr 2;15(4):e0226863. doi: 10.1371/journal.pone.0226863 (PMC7117680; doi:10.1371/journal.pone.0226863)
Supplement: S6 Table — DNA was extracted from Lymantria dispar legs or antennae using a Qiagen DNA extraction column and a field-ready protocol using Edwards buffer. DNA amplification was conducted in triplicate by qPCR using field-ready lyophilized reagents and fresh reagents. Average Ct values and standard deviations are reported for each of the conditions tested with a multiplex assay targeting the Asian and North American allele at the FS1 locus. The FS1 Asian allele carries the FAM fluorophore and the FS1 North American allele carries the CY5 fluorophore. NA = No Amplification. (DOCX) [file pone.0226863.s006.docx]

**S6 Table. Real-time PCR amplification of *Lymantria dispar* from adult legs and antennae.** DNA was extracted from *Lymantria dispar* legs or antennae a Qiagen DNA extraction column and a field-ready protocol using Edwards buffer. DNA amplification was conducted in triplicate by qPCR using field-ready lyophilized reagents and fresh reagents. Average C_t_ values and standard deviations are reported for each of the conditions tested with a multiplex assay targeting the Asian and North American allele at the FS1 locus. The FS1 Asian allele carries the FAM fluorophore and the FS1 North American allele carries the CY5 fluorophore. NA = No Amplification.

| **Material** | **Extraction** | **Target allele** | **Reagents** | **C_t_ values** | **Standard dev.** | **Rep.** |
| --- | --- | --- | --- | --- | --- | --- |
| Single leg of *L. dispar dispar* | Column | Asian | Lyophilized | NA | NA | 3 |
|  |  | North American |  | 36.24 | 0.55 |  |
|  | Edwards buffer | Asian | Fresh | NA | NA | 3 |
|  |  | North American |  | 31.92 | 0.30 |  |
|  | Column | Asian |  | NA | NA | 3 |
|  |  | North-American |  | 35.88 | 0.84 |  |
| Single leg of *L. dispar asiatica* | Edwards buffer | Asian | Lyophilized | 28.04 | 0.05 | 3 |
|  |  | North American |  | NA | NA |  |
|  | Column | Asian | Fresh | 29.03 | 0.18 | 3 |
|  |  | North American |  | NA | NA |  |
|  | Edwards buffer | Asian allele |  | 27.54 | 0.22 | 3 |
|  |  | North American |  | NA | NA |  |
| Pair of antennae from specimen #1 from pheromone trap | Edwards buffer | Asian | Lyophilized | NA | NA | 3 |
|  |  | North American |  | 30.99 | 0.16 |  |
|  |  | Asian | Fresh | NA | NA | 3 |
|  |  | North-American |  | 30.75 | 0.23 |  |
| Pair of antennae from specimen #2 from pheromone trap |  | Asian allele | Lyophilized | NA | NA | 3 |
|  |  | North American |  | 28.35 | 0.21 |  |
|  |  | Asian | Fresh | NA | NA | 3 |
|  |  | North American |  | 27.98 | 0.05 |  |
