# Supplementary material for: In Situ Processing and Efficient Environmental Detection (iSPEED) of tree pests and pathogens using point-of-use real-time PCR
Source: PLoS One. 2020 Apr 2;15(4):e0226863. doi: 10.1371/journal.pone.0226863 (PMC7117680; doi:10.1371/journal.pone.0226863)
Supplement: S7 Table — DNA was extracted from Cronartium comandra and C. ribicola spores using a Qiagen DNA extraction column and a field-ready protocol using Edwards buffer. DNA amplification was conducted in triplicate by qPCR using field-ready lyophilized reagents and fresh reagents. Average Ct values and standard deviations are reported for each condition tested with the Cronartium assays. The C. ribicola probe carries the FAM fluorophore and the C. comandrae carries the CY5 fluorophore. (DOCX) [file pone.0226863.s007.docx]

**S7 Table. Real-time PCR results for *Cronartium* spp spores.** DNA was extracted from *Cronartium comandrae* and *C. ribicola* spores using a Qiagen DNA extraction column and a field-ready protocol using Edwards buffer. DNA amplification was conducted in triplicate by qPCR using field-ready lyophilized reagents and fresh reagents. Average C_t_ values and standard deviations are reported for each condition tested with the *Cronartium* assays. The *C. ribicola* probe carries the FAM fluorophore and the *C. comandrae* carries the CY5 fluorophore.

| **Material** | **Extraction** | **Reagents** | **C_t_ values** | **Standard dev.** | **Rep.** |
| --- | --- | --- | --- | --- | --- |
| Single *C. comandrae* blister #1 | Column | Lyophilized | 27.87 | 1.30 | 3 |
|  |  | Fresh | 27.65 | 0.08 | 3 |
|  | Edwards buffer | Lyophilized | 28.56 | 0.26 | 3 |
|  |  | Fresh | 29.33 | 0.78 | 3 |
| Single *C. comandrae* blister #2 | Column | Lyophilized | 27.18 | 0.90 | 3 |
|  |  | Fresh | 26.53 | 0.29 | 3 |
|  | Edwards buffer | Lyophilized | 29.03 | 0.35 | 3 |
|  |  | Fresh | 29.66 | 0.35 | 3 |
| Pooled *C. ribicola* aeciospores | Column #1 | Lyophilized | 31.77 | 0.45 | 3 |
|  |  | Fresh | 31.27 | 0.11 | 3 |
|  | Column #2 | Lyophilized | 27.92 | 0.29 | 3 |
|  |  | Fresh | 28 | 0.18 | 3 |
|  | Edwards buffer #1 | Lyophilized | 32.23 | 0.56 | 2 |
|  |  | Fresh | 32.48 | 0.21 | 3 |
|  | Edwards buffer #2 | Lyophilized | 32.11 | 0.09 | 3 |
|  |  | Fresh | 32.96 | 0.10 | 3 |
