# Supplementary material for: In Situ Processing and Efficient Environmental Detection (iSPEED) of tree pests and pathogens using point-of-use real-time PCR
Source: PLoS One. 2020 Apr 2;15(4):e0226863. doi: 10.1371/journal.pone.0226863 (PMC7117680; doi:10.1371/journal.pone.0226863)
Supplement: S8 Table — Average Ct values obtained using the Cronartium assays on DNA extracted from C. ribicola aeciospores in the Franklin instrument. The C. ribicola probe carries the FAM fluorophore. (DOCX) [file pone.0226863.s008.docx]

**S8 Table. Real-time PCR results using the portable real-time PCR instrument.** Average C_t_ values obtained using the Cronartium assays on DNA extracted from *C. ribicola* aeciospores in the Franklin instrument. The *C. ribicola* probe carries the FAM fluorophore.

| **Material** | **Instrument** | **Extraction** | **Target** | **C_t_ values** | **Standard dev.** | **Rep.** |
| --- | --- | --- | --- | --- | --- | --- |
| Aeciospores #1 | Franklin | Column | *C. ribicola* | 26.92 | 0.92 | 2 |
| Aeciospores #2 |  | Edwards buffer |  | 33.48 | 0.28 | 2 |
| Aeciospores #3 |  |  |  | 31.94 | 0.64 | 2 |
