# Supplementary material for: In Situ Processing and Efficient Environmental Detection (iSPEED) of tree pests and pathogens using point-of-use real-time PCR
Source: PLoS One. 2020 Apr 2;15(4):e0226863. doi: 10.1371/journal.pone.0226863 (PMC7117680; doi:10.1371/journal.pone.0226863)
Supplement: S9 Table — Average Ct values obtained using the Cronartium assays with C. ribicola and C. comandrae DNA. One hybrid was tested as well. A) results obtained with the assays used as simplex; B) results obtained with the assays used as a duplex. The C. ribicola probe carries the FAM fluorophore and the C. comandrae probe carries the HEX fluorophore. NA = No Amplification. (DOCX) [file pone.0226863.s009.docx]

**S9 Table. Specificity tests for the *Cronartium* assays.** Average C_t_ values obtained using the *Cronartium* assays with *C. ribicola* and *C. comandrae* DNA. One hybrid was tested as well. A) results obtained with the assays used as simplex; B) results obtained with the assays used as a duplex. The *C. ribicola* probe carries the FAM fluorophore and the *C. comandrae* probe carries the HEX fluorophore. NA = No Amplification.

**A)**

| **Sample** | **Species** | ***C. ribicola*** | | ***C. comandrae*** | | **Rep.** |
| --- | --- | --- | --- | --- | --- | --- |
|  |  | **C_t_ values** | **Standard dev.** | **C_t_ values** | **Standard dev.** |  |
| CR-BM-1.3 | *C. ribicola* | 25.3 | 0.03 | NA | NA | 2 |
| CR-BM-1.8 |  | 24.6 | 0.08 | NA | NA | 2 |
| CR-BM-2.1 |  | 28.4 | 0.18 | NA | NA | 2 |
| CR-BM-3.4 |  | 30.8 | 0.12 | NA | NA | 2 |
| CR-BM-3.61 |  | 23.2 | 0.03 | NA | NA | 2 |
| CS-Sm1 |  | 29.9 | 0.06 | NA | NA | 2 |
| PG-CC-C3.3 | *C. comandrae* | NA | NA | 27.0 | 0.06 | 2 |
| SM-CC-1B |  | NA | NA | 31.7 | 0.02 | 2 |
| SM-CC-2A |  | NA | NA | 28.3 | 0.11 | 2 |
| SM-CC-2C |  | NA | NA | 28.1 | 0.08 | 2 |
| PS2Q | Hybrid | 24.0 | 0.22 | 26.3 | 0.14 | 2 |

**B)**

| **Sample** | **Species** | ***C. ribicola*** | | ***C. comandrae*** | | **Rep.** |
| --- | --- | --- | --- | --- | --- | --- |
|  |  | **C_t_ values** | **Standard dev.** | **C_t_ values** | **Standard dev.** |  |
| CR-BM-1.3 | *C. ribicola* | 25.1 | 0.04 | NA | NA | 2 |
| CR-BM-1.8 |  | 25.1 | 0.41 | NA | NA | 2 |
| CR-BM-2.1 |  | 28.3 | 0.09 | NA | NA | 2 |
| CR-BM-3.4 |  | 31.3 | 0.24 | NA | NA | 2 |
| CR-BM-3.61 |  | 23.3 | 0.01 | NA | NA | 2 |
| CS-Sm1 |  | 29.2 | 0.19 | NA | NA | 2 |
| PG-CC-C3.3 | *C. comandrae* | NA | NA | 27.6 | 0.06 | 2 |
| SM-CC-1B |  | NA | NA | 32.0 | 0.03 | 2 |
| SM-CC-2A |  | NA | NA | 28.9 | 0.01 | 2 |
| SM-CC-2C |  | NA | NA | 28.5 | 0.03 | 2 |
| PS2Q | Hybrid | 26.8 | 0.08 | 24.1 | 0.15 | 2 |
