# Supplementary material for: In Situ Processing and Efficient Environmental Detection (iSPEED) of tree pests and pathogens using point-of-use real-time PCR
Source: PLoS One. 2020 Apr 2;15(4):e0226863. doi: 10.1371/journal.pone.0226863 (PMC7117680; doi:10.1371/journal.pone.0226863)
Supplement: S1 Data — (DOCX) [file pone.0226863.s010.docx]

**Supplemental material and method**

The *Cronartium* DNA samples used for the specificity and cross-reactivity tests are described in Allen, 2019 [5]. The species identity or hybrid status was confirmed by morphology and DNA sequencing. Real-time PCR was performed on a Viia7 real-time PCR instrument (ThermoFisher Scientific, Waltham, MA, USA), using the QuantiFast Probe PCR master mix (Qiagen, Venlo, Netherlands). The simplex reactions were prepared as such: 0.5 µM forward and reverse primers, 0.4 µM probe, 1x master mix. 1 µL of DNA was used with a final volume of 10 µL. The duplex reactions were prepared as follows: 0.5 µM forward and reverse primers, 0.4 µM probes, 1x master mix. 1 µL of DNA was used with a final volume of 10 µL. The cycling conditions were 95°C for 5 min, 45 cycles of 95°C for 30 sec and 60°C for 30 sec. Results are reported in Table S9.

**Standard Operating Procedure for point-of-use real-time PCR**

**Material:**

- Portable thermocycler Franklin (Biomeme)

- 100 µL PCR tube strips and caps (Applied biosystems, ref 4358293 and ref 432032)

- 20 µL Pastettes (Alpha laboratories, ref LW4730-500)

- 5 mL screw cap tubes (Axygen, SCT-5ML-S)

- Tools to prepare the samples (tweezers, scalpel, punch)

- Tris base (Fisher Scientific, ref BP152-1)

- Ethylenediamine Tetraacetic Acid, Disodium Salt Dihydrate (EDTA) (Fisher Scientific, ref BP120-1)

- NaCL (Fisher Scientific, ref BP358-1)

- Sodium Dodecyl Sulfate (SDS) (Bioshop, ref SDS001-500)

- PolyVinylPolyPirrolidone (PVPP) (Sigma-Aldrich, ref 77627-100G)

- D-(+)-Trehalose dihydrate (Sigma-Aldrich, ref T9531-5G)

- Water (Sigma-Aldrich, ref W4502)

- Mineral Oil (Ward’s Science, ref 470108-800)

- QuantiTect Multiplex PCR, noROX kit (Qiagen, ref 204743)

- Suitable TaqMan assays (various manufacturers)

Solutions to prepare:

- Edwards: Tris 200 mM pH 8.0, EDTA 25 mM, NaCl 250 mM, SDS 0.5% (w/v) (+ PVPP 1% (w/v) for plant samples)

- Trehalose 30% (w/v)

- Water aliquots for DNA dilution in 5 mL screw-caps tube (980 µL or 1980 µL)

**Method:**

1 Prepare lyophilized reactions

- Mix 10 uL Quantitect mastermix with primers and probe (200-500 nM each)

- Add trehalose 30% to reach 5% final

- Freeze to -20°C

- Lyophilize in the dark for 60-90 min

2 DNA extraction/real-time PCR setup

- Depending on sample, place 2.5-20mg of material in an empty 100 µL PCR tube

- Add 40 µL of Edwards buffer to the tube with a clean Pastette, cap and incubate for 10 min at 95°C in the portable thermocycler

- Dilute 20 µL the DNA extract in pre-aliquoted water in the 5 mL screw-cap tube (980 µL for 1:50, 1980 µL for 1:100) with a clean Pastette

- Mix and distribute 20 µL of the diluted extract to a freeze-dried reaction with a clean Pastette

- Add 40 µL of mineral oil and close the tube with a clean Pastette

3 real-time PCR

- run the reactions with the following conditions:

95°C for 15 min

40 cycles of:

95°C for 15 sec

60°C for 1min30

**Reference**

1. [Feau N, Ojeda DI, Beauseigle S, Bilodeau GJ, Brar A, Cervantes‐Arango S, et al. Improved detection and identification of the sudden oak death pathogen Phytophthora ramorum and the Port Orford cedar root pathogen *Phytophthora lateralis*. Plant Pathol. 2019;68(5):878–88.](https://www.zotero.org/google-docs/?iBomEe)
2. [Bilodeau GJ, Martin FN, Coffey MD, Blomquist CL. Development of a Multiplex Assay for Genus- and Species-Specific Detection of *Phytophthora* Based on Differences in Mitochondrial Gene Order. Phytopathology. 2014 Jul;104(7):733–48.](https://www.zotero.org/google-docs/?iBomEe)
3. [Stewart D, Zahiri R, Djoumad A, Freschi L, Lamarche J, Holden D, et al. A Multi-Species TaqMan PCR Assay for the Identification of Asian Gypsy Moths (*Lymantria* spp.) and Other Invasive Lymantriines of Biosecurity Concern to North America. PloS One. 2016;11(8):e0160878.](https://www.zotero.org/google-docs/?iBomEe)
4. [Herath P, Beauseigle S, Dhillon B, Ojeda DI, Bilodeau G, Isabel N, et al. Anthropogenic signature in the incidence and distribution of an emerging pathogen of poplars. Biol Invasions. 2016 Apr;18(4):1147–1161.](https://www.zotero.org/google-docs/?iBomEe)
5. Allen K. Evaluating the presence and introgression of the hybrid forest pathogen *Cronartium x flexili.* M. Sc. Thesis, The University of British Columbia. 2019. Available from https://dx.doi.org/10.14288/1.0380539
