# Supplementary material for: In Situ Processing and Efficient Environmental Detection (iSPEED) of tree pests and pathogens using point-of-use real-time PCR
Source: PLoS One. 2020 Apr 2;15(4):e0226863. doi: 10.1371/journal.pone.0226863 (PMC7117680; doi:10.1371/journal.pone.0226863)
Supplement: Attachment — Submitted filename: Supporting Information_AE_tracked_changes.docx [file pone.0226863.s013.docx]

**S1 Table. Oligonucleotide sequences used in qPCR assays.** The table lists all the oligonucleotides used in the various tests. Probes were ordered with a FAM fluorophore for simplex reactions and with the FAM and CY5 combination for duplexes. The + sign denotes a LNA base.

| **Name** | **Type** | **Assay** | **Sequence** | **Reference** |
| --- | --- | --- | --- | --- |
| Pram-C62-F | Primer | *P. ramorum*, TAIGA-C62  nuclear | AACATGCTCGTGCTCAAGTG | [(1)](https://www.zotero.org/google-docs/?wNfyLp) |
| Pram-C62-R | Primer |  | CGGTGTTCTGGCGTTCTAGT |  |
| Pram-C62-P | Probe |  | CAAGGGGACCGGAACCGTAT |  |
| Phyto_gen_Fflap | Primer | *P. ramorum*, *TrnM*  mitochondrial | AATAAATCATAACCTTCTTTACAACAAGAATTAATGAG | [(2)](https://www.zotero.org/google-docs/?DaC4Vg) |
| Pram_Rflap | Primer |  | AATAAATCATAATATAGGTAAAATTTGTAATAAATGTTGACT |  |
| Pram_nad9sp_1F | Probe |  | ACGTTACGTCTAGACTTGTATTATGCATTG |  |
| FS1 F2-16 | Primer | *Lymantria dispar* | GATGGTGGGTGTCGT | [(3)](https://www.zotero.org/google-docs/?sHOGPM) |
| FS1 R176-200 (71–95) | Primer |  | GATTCATCTGATCCTGATAATTCAT |  |
| FS1 AGM 62–86 | Probe |  | ACTCAACATAAAGTATGCCAACTCG |  |
| FS1 EGM RC 21–47 | Probe |  | AGTACTGCTGTATACATTTTAAACGTC |  |
| Cronartium Dcon10 1 F | Primer | *C. comandrae* | AAAGCACTCACCCTTCGGTC | This work, unpublished results |
| Cronartium Dcon10 1 R | Primer |  | GACTGGACACATTGGCTCCTT |  |
| Cribicola Dcon10 1 | Probe |  | TGGCC+T+C+G+ACGTGT |  |
| Ccomandrae Dcon10 1 | Probe |  | TGGC+C+T+T+C+ACGTGT |  |
| Smus_SSU193F | Primer | *S. musiva* | CGGTATTTTCAGCCTGCAG | [(4)](https://www.zotero.org/google-docs/?LmlBLl) |
| Smus_SSU_288R | Primer |  | GCCGTTATCCGTACAACTGA |  |
| Smus_SSU243F_probe | Probe |  | AGGTAGATATTAGGACAATTGGTGTAAAGATGAT |  |
| rbcL_delto-1F | Primer | *Populus spp.* | CACCCTTGGGGAAATGC |  |
| rbcL_delto-1R | Primer |  | CTTCACGGATAATTTCATTACCCTC |  |
| rbcL_delto_probe | Probe |  | CGAGTAGCTCTAGAAGCATGTGTACAAGCT |  |

**S2 Table. Reagents used in fresh reactions.** The table lists the reagents used to prepare the fresh reactions for each assay.

| **Target organism** | **Reagent** | **Amount** |
| --- | --- | --- |
| *Sphaerulina musiva* | DNA template | 2 µL |
|  | QuantiTect mastermix | 10 µL |
|  | Primer 10 µM, each | 0.8 µL |
|  | Probe 100 µM | 4 µL |
|  | Trehalose 30% | 1.13 µL |
|  | Water | 1.27 µL |
|  | Total volume | 20 µL |
| *Cronartium* spp.^1^ | DNA template | 2 µL |
|  | QuantiTect mastermix | 10 µL |
|  | 20x Primers/probe mix | 1 µL |
|  | Trehalose 30% | 1.22 µL |
|  | Water | 4.78 µL |
|  | Total volume | 20 µL |
| *Phytophthora ramorum*^2^ | DNA template | 2 µL |
|  | QuantiTect mastermix | 10 µL |
|  | 20x Primers/probe mix | 1 µL |
|  | Trehalose 30% | 1.22 µL |
|  | Water | 5.78 µL |
|  | Total volume | 20 µL |
| *Lymantria dispar* | DNA template | 2 µL |
|  | QuantiTect mastermix | 10 µL |
|  | Primer 100 µM, each | 0.1 µL |
|  | Probe 100 µM, each | 0.04 µL |
|  | Trehalose 30% | 1.14 µL |
|  | Water | 6.58 µL |
|  | Total volume | 20 µL |
| ^1^The 20x Primers/probe mix is prepared to 5 µM for each primer and 2 µM for each probe | | |
| ^2^The 20x Primers/probe mix is prepared to 4 µM for each primer and the probe | | |

**S3 Table. Reagents used in lyophilized reactions.** The table lists the reagents used to prepare the lyophilized strips for each assay.

| **Target organism** | **Reagent** | **Amount** |
| --- | --- | --- |
| *Sphaerulina musiva* | QuantiTect mastermix | 10 µL |
|  | Primer 100 µM, each | 0.08 µL |
|  | Probe 100 µM | 0.04 µL |
|  | Trehalose 30%^1^ | 1.13 µL |
|  | Total volume | 11.33 µL |
| *Cronartium* spp.^2^ | QuantiTect mastermix | 10 µL |
|  | 20x Primers/probe mix | 1 µL |
|  | Trehalose 30%^1^ | 1.22 µL |
|  | Total volume | 12.22 µL |
| *Phytophthora ramorum*^3^ | QuantiTect mastermix | 10 µL |
|  | 20x Primers/probe mix | 1 µL |
|  | Trehalose 30%^1^ | 1.22 µL |
|  | Volume | 12.22 µL |
| *Lymantria dispar* | QuantiTect mastermix | 10 µL |
|  | Primer 100 µM, each | 0.1 µL |
|  | Probe 100 µM, each | 0.04 µL |
|  | Trehalose 30%^1^ | 1.14 µL |
|  | Total volume | 11.42 µL |
| ^1^For the reactions without trehalose, the 30% trehalose is omitted and the total aliquoted volume is adjusted accordingly. | | |
| ^2^The 20x Primers/probe mix is prepared to 5 µM for each primer and 2 µM for each probe. | | |
| ^3^The 20x Primers/probe mix is prepared to 4 µM for each primer and the probe | | |

**S4 Table. Real-time PCR amplification of *Sphaerulina musiva* directly from naturally-infected poplar hybrid leaves.** DNA was extracted from leaves of poplar hybrids (*P. trichocarpa* x *P. deltoides)* infected by *S. musiva* using a Qiagen DNA extraction column and a field-ready protocol using Edwards buffer. DNA amplification was conducted in triplicate by qPCR using field-ready lyophilized reagents and fresh reagents. Average C_t_ values of the replicates are reported for each of the conditions tested with the plant internal control (RbcL) and the *S. musiva* (SepMu) assays. All tests were conducted using material from the same leaf disc to allow direct comparisons between extraction methods. Both probes used carry the FAM fluorophore.

| **Target** | **Extraction** | **Reagents** | **C_t_ values** | **Standard dev.** | **Rep.** |
| --- | --- | --- | --- | --- | --- |
| RbcL | Column | Lyophilized | 17.52 | 0.03 | 3 |
|  |  | Fresh | 17.3 | 0.11 | 3 |
|  | Edward buffer | Lyophilized | 21.24 | 0.07 | 3 |
|  |  | Fresh | 21.46 | 0.08 | 3 |
| SepMu | Column | Lyophilized | 23.8 | 0.42 | 3 |
|  |  | Fresh | 23.34 | 0.07 | 3 |
|  | Edward buffer | Lyophilized | 25.12 | 0.02 | 3 |
|  |  | Fresh | 25.41 | 0.09 | 3 |

**S5 Table. Real-time PCR amplification of *Phytophthora ramorum* from artificially infected *Rhododendron* leaves.** DNA was extracted from Rhododendron leaves using a Qiagen DNA extraction column and a field-ready protocol using Edwards buffer. A pure culture of *P. ramorum* was also extracted with a Qiagen extraction protocol as a positive control. DNA amplification was conducted in triplicate by qPCR using field-ready lyophilized reagents and fresh reagents. Average C_t_ values of the replicates are reported for each of the conditions tested with mitochondrial and nuclear *P. ramorum* assays. All tests were conducted using material from the same leaf disc. Both probes used carry the FAM fluorophore. NA = No Amplification.

| **Material** | **Extraction** | **Target** | **Reagents** | **C_t_ values** | **Standard dev.** | **Rep.** |
| --- | --- | --- | --- | --- | --- | --- |
| Culture | Column | *TrnM* mitochondrial | Fresh | 19.18 | 0.04 | 3 |
| *R. BCHM* | Edwards buffer |  |  | NA | NA | 3 |
| *R. macrophyllum* |  |  |  | NA | NA | 3 |
| *R. purdomii* |  |  |  | NA | NA | 3 |
| *R. BCHM*, inoculated |  |  |  | 32.16 | 0.05 | 3 |
| *R. macrophyllum*, inoculated |  |  |  | 28.16 | 0.44 | 3 |
| *R. purdomii*, inoculated |  |  |  | 26.64 | 0.07 | 3 |
| Culture | Column |  | Lyophilized | 15.77 | 0.13 | 3 |
| *R. BCHM* | Edwards buffer |  |  | NA | NA | 3 |
| *R. macrophyllum* |  |  |  | NA | NA | 3 |
| *R. purdomii* |  |  |  | NA | NA | 3 |
| *R. BCHM*, inoculated |  |  |  | 29.12 | 0.09 | 3 |
| *R. macrophyllum*, inoculated |  |  |  | 27.23 | 0.09 | 3 |
| *R. purdomii*, inoculated |  |  |  | 26.03 | 0.19 | 3 |
| Culture | Column | TAIGA-C62  Nuclear | Fresh | 25.80 | 0.10 | 3 |
| *R. BCHM* | Edwards buffer |  |  | NA | NA | 3 |
| *R. macrophyllum* |  |  |  | NA | NA | 3 |
| *R. purdomii* |  |  |  | NA | NA | 3 |
| *R. BCHM*, inoculated |  |  |  | 34.62 | 0.48 | 3 |
| *R. macrophyllum*, inoculated |  |  |  | 33.40 | 0.31 | 3 |
| *P. purdomii*, inoculated |  |  |  | 34.24 | 0.32 | 3 |
| Culture | Column |  | Lyophilized | 21.93 | 0.55 | 3 |
| *R. BCHM* | Edwards buffer |  |  | NA | NA | 3 |
| *R. macrophyllum* |  |  |  | NA | NA | 3 |
| *R. purdomii* |  |  |  | NA | NA | 3 |
| *R. BCHM*, inoculated |  |  |  | 33.48 | 0.07 | 3 |
| *R. macrophyllum*, inoculated |  |  |  | 33.20 | 0.24 | 3 |
| *R. purdomii*, inoculated |  |  |  | 33.65 | 0.26 | 3 |

**S6 Table. Real-time PCR amplification of *Lymantria dispar* from adult legs and antennae.** DNA was extracted from *Lymantria dispar* legs or antennae a Qiagen DNA extraction column and a field-ready protocol using Edwards buffer. DNA amplification was conducted in triplicate by qPCR using field-ready lyophilized reagents and fresh reagents. Average C_t_ values and standard deviations are reported for each of the conditions tested with a multiplex assay targeting the Asian and North American allele at the FS1 locus. The FS1 Asian allele carries the FAM fluorophore and the FS1 North American allele carries the CY5 fluorophore. NA = No Amplification.

| **Material** | **Extraction** | **Target allele** | **Reagents** | **C_t_ values** | **Standard dev.** | **Rep.** |
| --- | --- | --- | --- | --- | --- | --- |
| Single leg of *L. dispar dispar* | Column | Asian | Lyophilized | NA | NA | 3 |
|  |  | North-American |  | 36.24 | 0.55 |  |
|  | Edwards buffer | Asian | Fresh | NA | NA | 3 |
|  |  | North-American |  | 31.92 | 0.30 |  |
|  | Column | Asian |  | NA | NA | 3 |
|  |  | North-American |  | 35.88 | 0.84 |  |
| Single leg of *L. dispar asiatica* | Edwards buffer | Asian | Lyophilized | 28.04 | 0.05 | 3 |
|  |  | North-American |  | NA | NA |  |
|  | Column | Asian | Fresh | 29.03 | 0.18 | 3 |
|  |  | North-American |  | NA | NA |  |
|  | Edwards buffer | Asian allele |  | 27.54 | 0.22 | 3 |
|  |  | North-American |  | NA | NA |  |
| Pair of antennae from specimen #1 from pheromone trap | Edwards buffer | Asian | Lyophilized | NA | NA | 3 |
|  |  | North-American |  | 30.99 | 0.16 |  |
|  |  | Asian | Fresh | NA | NA | 3 |
|  |  | North-American |  | 30.75 | 0.23 |  |
| Pair of antennae from specimen #2 from pheromone trap |  | Asian allele | Lyophilized | NA | NA | 3 |
|  |  | North-American |  | 28.35 | 0.21 |  |
|  |  | Asian | Fresh | NA | NA | 3 |
|  |  | North-American |  | 27.98 | 0.05 |  |

**S7 Table. Real-time PCR results for *Cronartium* spp spores.** DNA was extracted from *Cronartium comandrae* and *C. ribicola* spores using a Qiagen DNA extraction column and a field-ready protocol using Edwards buffer. DNA amplification was conducted in triplicate by qPCR using field-ready lyophilized reagents and fresh reagents. Average C_t_ values and standard deviations are reported for each condition tested with the *Cronartium* assays. The *C. ribicola* probe carries the FAM fluorophore and the *C. comandrae* carries the CY5 fluorophore.

| **Material** | **Extraction** | **Reagents** | **C_t_ values** | **Standard dev.** | **Rep.** |
| --- | --- | --- | --- | --- | --- |
| Single *C. comandrae* blister #1 | Column | Lyophilized | 27.87 | 1.30 | 3 |
|  |  | Fresh | 27.65 | 0.08 | 3 |
|  | Edwards buffer | Lyophilized | 28.56 | 0.26 | 3 |
|  |  | Fresh | 29.33 | 0.78 | 3 |
| Single *C. comandrae* blister #2 | Column | Lyophilized | 27.18 | 0.90 | 3 |
|  |  | Fresh | 26.53 | 0.29 | 3 |
|  | Edwards buffer | Lyophilized | 29.03 | 0.35 | 3 |
|  |  | Fresh | 29.66 | 0.35 | 3 |
| Pooled *C. ribicola* aeciospores | Column #1 | Lyophilized | 31.77 | 0.45 | 3 |
|  |  | Fresh | 31.27 | 0.11 | 3 |
|  | Column #2 | Lyophilized | 27.92 | 0.29 | 3 |
|  |  | Fresh | 28 | 0.18 | 3 |
|  | Edwards buffer #1 | Lyophilized | 32.23 | 0.56 | 2 |
|  |  | Fresh | 32.48 | 0.21 | 3 |
|  | Edwards buffer #2 | Lyophilized | 32.11 | 0.09 | 3 |
|  |  | Fresh | 32.96 | 0.10 | 3 |

**S8 Table. Real-time PCR results using the portable real-time PCR instrument.** Average C_t_ values obtained using the Cronartium assays on DNA extracted from *C. ribicola* aeciospores in the Franklin instrument. The *C. ribicola* probe carries the FAM fluorophore.

| **Material** | **Instrument** | **Extraction** | **Target** | **C_t_ values** | **Standard dev.** | **Rep.** |
| --- | --- | --- | --- | --- | --- | --- |
| Aeciospores #1 | Franklin | Column | *C. ribicola* | 26.92 | 0.92 | 2 |
| Aeciospores #2 |  | Edwards buffer |  | 33.48 | 0.28 | 2 |
| Aeciospores #3 |  |  |  | 31.94 | 0.64 | 2 |

**S9 Table. Specificity tests for the *Cronartium* assays.** Average C_t_ values obtained using the *Cronartium* assays with *C. ribicola* and *C. comandrae* DNA. One hybrid was tested as well. A) results obtained with the assays used as simplex; B) results obtained with the assays used as a duplex. The *C. ribicola* probe carries the FAM fluorophore and the *C. comandrae* probe carries the HEX fluorophore. NA = No Amplification.

**A)**

| **Sample** | **Species** | ***C. ribicola*** | | ***C. comandrae*** | | **Rep.** |
| --- | --- | --- | --- | --- | --- | --- |
|  |  | **C_t_ values** | **Standard dev.** | **C_t_ values** | **Standard dev.** |  |
| CR-BM-1.3 | *C. ribicola* | 25.3 | 0.03 | NA | NA | 2 |
| CR-BM-1.8 |  | 24.6 | 0.08 | NA | NA | 2 |
| CR-BM-2.1 |  | 28.4 | 0.18 | NA | NA | 2 |
| CR-BM-3.4 |  | 30.8 | 0.12 | NA | NA | 2 |
| CR-BM-3.61 |  | 23.2 | 0.03 | NA | NA | 2 |
| CS-Sm1 |  | 29.9 | 0.06 | NA | NA | 2 |
| PG-CC-C3.3 | *C. comandrae* | NA | NA | 27.0 | 0.06 | 2 |
| SM-CC-1B |  | NA | NA | 31.7 | 0.02 | 2 |
| SM-CC-2A |  | NA | NA | 28.3 | 0.11 | 2 |
| SM-CC-2C |  | NA | NA | 28.1 | 0.08 | 2 |
| PS2Q | Hybrid | 24.0 | 0.22 | 26.3 | 0.14 | 2 |

**B)**

| **Sample** | **Species** | ***C. ribicola*** | | ***C. comandrae*** | | **Rep.** |
| --- | --- | --- | --- | --- | --- | --- |
|  |  | **C_t_ values** | **Standard dev.** | **C_t_ values** | **Standard dev.** |  |
| CR-BM-1.3 | *C. ribicola* | 25.1 | 0.04 | NA | NA | 2 |
| CR-BM-1.8 |  | 25.1 | 0.41 | NA | NA | 2 |
| CR-BM-2.1 |  | 28.3 | 0.09 | NA | NA | 2 |
| CR-BM-3.4 |  | 31.3 | 0.24 | NA | NA | 2 |
| CR-BM-3.61 |  | 23.3 | 0.01 | NA | NA | 2 |
| CS-Sm1 |  | 29.2 | 0.19 | NA | NA | 2 |
| PG-CC-C3.3 | *C. comandrae* | NA | NA | 27.6 | 0.06 | 2 |
| SM-CC-1B |  | NA | NA | 32.0 | 0.03 | 2 |
| SM-CC-2A |  | NA | NA | 28.9 | 0.01 | 2 |
| SM-CC-2C |  | NA | NA | 28.5 | 0.03 | 2 |
| PS2Q | Hybrid | 26.8 | 0.08 | 24.1 | 0.15 | 2 |

**Supplemental material and method**

The *Cronartium* DNA samples used for the specificity and cross-reactivity tests are described in Allen, 2019 [5]. The species identity or hybrid status was confirmed by morphology and DNA sequencing. Real-time PCR was performed on a Viia7 real-time PCR instrument (ThermoFisher Scientific, Waltham, MA, USA), using the QuantiFast Probe PCR master mix (Qiagen, Venlo, Netherlands). The simplex reactions were prepared as such: 0.5 µM forward and reverse primers, 0.4 µM probe, 1x master mix. 1 µL of DNA was used with a final volume of 10 µL. The duplex reactions were prepared as follows: 0.5 µM forward and reverse primers, 0.4 µM probes, 1x master mix. 1 µL of DNA was used with a final volume of 10 µL. The cycling conditions were 95°C for 5 min, 45 cycles of 95°C for 30 sec and 60°C for 30 sec. Results are reported in Table S9.

**Standard Operating Procedure for point-of-use real-time PCR**

**Material:**

-	Portable thermocycler Franklin (Biomeme)

-	100 µL PCR tube strips and caps (Applied biosystems, ref 4358293 and ref 432032)

-	20 µL Pastettes (Alpha laboratories, ref LW4730-500)

-	5 mL screw cap tubes (Axygen, SCT-5ML-S)

-	Tools to prepare the samples (tweezers, scalpel, punch)

-	Tris base (Fisher Scientific, ref BP152-1)

-	Ethylenediamine Tetraacetic Acid, Disodium Salt Dihydrate (EDTA) (Fisher Scientific, ref BP120-1)

-	NaCL (Fisher Scientific, ref BP358-1)

-	Sodium Dodecyl Sulfate (SDS) (Bioshop, ref SDS001-500)

-	PolyVinylPolyPirrolidone (PVPP) (Sigma-Aldrich, ref 77627-100G)

-	D-(+)-Trehalose dihydrate (Sigma-Aldrich, ref T9531-5G)

-	Water (Sigma-Aldrich, ref W4502)

-	Mineral Oil (Ward’s Science, ref 470108-800)

-	QuantiTect Multiplex PCR, noROX kit (Qiagen, ref 204743)

-	Suitable TaqMan assays (various manufacturers)

Solutions to prepare:

-	Edwards: Tris 200 mM pH 8.0, EDTA 25 mM, NaCl 250 mM, SDS 0.5% (w/v) (+ PVPP 1% (w/v) for plant samples)

-	Trehalose 30% (w/v)

-	Water aliquots for DNA dilution in 5 mL screw-caps tube (980 µL or 1980 µL)

**Method:**

1	Prepare lyophilized reactions

-	Mix 10 uL Quantitect mastermix with primers and probe (200-500 nM each)

-	Add trehalose 30% to reach 5% final

-	Freeze to -20°C

-	Lyophilize in the dark for 60-90 min

2	DNA extraction/real-time PCR setup

-	Depending on sample, place 2.5-20mg of material in an empty 100 µL PCR tube

-	Add 40 µL of Edwards buffer to the tube with a clean Pastette, cap and incubate for 10 min at 95°C in the portable thermocycler

-	Dilute 20 µL the DNA extract in pre-aliquoted water in the 5 mL screw-cap tube (980 µL for 1:50, 1980 µL for 1:100) with a clean Pastette

-	Mix and distribute 20 µL of the diluted extract to a freeze-dried reaction with a clean Pastette

-	Add 40 µL of mineral oil and close the tube with a clean Pastette

3	real-time PCR

-	run the reactions with the following conditions:

95°C for 15 min

40 cycles of:

95°C for 15 sec

60°C for 1min30

**Reference**

1. [Feau N, Ojeda DI, Beauseigle S, Bilodeau GJ, Brar A, Cervantes‐Arango S, et al. Improved detection and identification of the sudden oak death pathogen Phytophthora ramorum and the Port Orford cedar root pathogen Phytophthora lateralis. Plant Pathology. 2019;68(5):878–88.](https://www.zotero.org/google-docs/?iBomEe)
2. [Bilodeau GJ, Martin FN, Coffey MD, Blomquist CL. Development of a multiplex assay for genus- and species-specific detection of Phytophthora based on differences in mitochondrial gene order. Phytopathology. 2014 Jul;104(7):733–48.](https://www.zotero.org/google-docs/?iBomEe)
3. [Stewart D, Zahiri R, Djoumad A, Freschi L, Lamarche J, Holden D, et al. A Multi-Species TaqMan PCR Assay for the Identification of Asian Gypsy Moths (*Lymantria spp.*) and Other Invasive Lymantriines of Biosecurity Concern to North America. PloS one. 2016;11(8):e0160878.](https://www.zotero.org/google-docs/?iBomEe)
4. [Herath P, Beauseigle S, Dhillon B, Ojeda DI, Bilodeau G, Isabel N, et al. Anthropogenic signature in the incidence and distribution of an emerging pathogen of poplars. Biological Invasions. 2016 Apr;18(4):1147–1161.](https://www.zotero.org/google-docs/?iBomEe)
5. Allen K. Evaluating the presence and introgression of the hybrid forest pathogen *Cronartium x flexil.* The University of British Columbia. 2019. Available from https://dx.doi.org/10.14288/1.0380539
